# Overcoming of Radioresistance in Non-small Cell Lung Cancer by microRNA-320a Through HIF1α-Suppression Mediated Methylation of PTEN

**DOI:** 10.3389/fcell.2020.553733

**Published:** 2020-11-10

**Authors:** Li-Ming Xu, Hao Yu, Ya-Jing Yuan, Jiao Zhang, Yue Ma, Xu-Chen Cao, Jun Wang, Lu-Jun Zhao, Ping Wang

**Affiliations:** ^1^Department of Radiotherapy, Tianjin Medical University Cancer Institute & Hospital, National Clinical Research Center for Cancer, Tianjin, China; ^2^Key Laboratory of Cancer Prevention and Therapy, Tianjin’s Clinical Research Center for Cancer, Tianjin, China; ^3^Key Laboratory of Breast Cancer Prevention and Therapy, Tianjin Medical University, Ministry of Education, Tianjin, China; ^4^Department of Radiotherapy, Tianjin Medical University Cancer Hospital Airport Hospital, Tianjin, China; ^5^Department of Anesthesia, Tianjin Medical University Cancer Institute & Hospital, National Clinical Research Center for Cancer, Tianjin, China; ^6^The First Department of Breast Cancer, Tianjin Medical University Cancer Institute & Hospital, National Clinical Research Center for Cancer, Tianjin, China

**Keywords:** non-small cell lung cancer, microRNA-320a, HIF1α, KDM5B, PTEN, radioresistance

## Abstract

**Background:**

Radioresistance is a major challenge in the use of radiotherapy for the treatment of lung cancer while microRNAs (miRs) have been reported to participate in multiple essential cellular processes including radiosensitization. This study was conducted with the main objective of investigating the potential role of miR-320a in radioresistance of non-small cell lung cancer (NSCLC) via the possible mechanism related to HIF1α, KDM5B, and PTEN.

**Methods:**

Firstly, NSCLC radiosensitivity-related microarray dataset GSE112374 was obtained. Then, the expression of miR-320a, HIF1α, KDM5B, and PTEN was detected in the collected clinical NSCLC samples, followed by Pearson’s correlation analysis. Subsequently, ChIP assay was conducted to determine the content of the PTEN promoter fragment enriched by the IgG antibody and H3K4me3 antibody. Finally, a series of *in vitro* and *in vivo* assays were performed in order to evaluate the effects of miR-320a on radioresistance of NSCLC with the involvement of HIF1α, KDM5B, and PTEN.

**Results:**

The microarray dataset GSE112374 presented with a high expression of miR-320a in NSCLC radiosensitivity samples, which was further confirmed in our clinical samples with the use of reverse transcription-quantitative polymerase chain reaction. Moreover, miR-320a negatively targeted HIF1α, inhibiting radioresistance of NSCLC. Interestingly, miR-320a suppressed the expression of KDM5B, and KDM5B was found to enhance the radioresistance of NSCLC through the downregulation of PTEN expression. The inhibition of miR-320a in radioresistance of NSCLC was also reproduced by *in vivo* assay.

**Conclusion:**

Taken together, our findings were suggestive of the inhibitory effect of miR-320a on radioresistance of NSCLC through HIF1α-suppression mediated methylation of PTEN.

## Introduction

Clinically, non-small cell lung cancers (NSCLCs) account for more than 85% of lung cancers and include adenocarcinomas, squamous-cell and large-cell carcinomas, with a 5-year survival rate of ∼20% (Gridelli et al., 2015; Mizuno et al., 2017). NSCLC is a kind of heterogeneous tumor and the risk factors for the development of this cancer include cooking oil vapor, smoke, exposure to asbestos/heavy metals, hormonal replacement therapy, albumin, gender, genetic susceptibility, and the serum creatinine concentration of thrombocytopenia (Jiang et al., 2013; Smolle and Pichler, 2019). Currently, the only available treatment for patients with stage III NSCLC is radiotherapy, which has an unsatisfactory outcome, with a median survival of 10 months and 5-year survival for only 5% patients (Dawe et al., 2016). Radioresistance, which occurs in some patients with NSCLC secondary to tumor heterogeneity in terms of the cell of origin, pathology, etiology, and molecular/genetic pathogenesis, has been the most challenging problem, as it leads to the failure of radiotherapy (Gomez-Casal et al., 2013). Moreover, the key molecules related to radiation-stimulated radioresistance remain poorly understood (Tang et al., 2016). Hence, the presence of these challenges in the treatment of NSCLC means that there is an urgent need for the development of a novel and effective biomarker to manage radioresistance of NSCLC.

Noticeably, microRNAs (miRs) which are small and non-coding RNAs, could mediate post-transcriptional gene expression, and have been reported to have a significant involvement in cancer radioresistance (Korpela et al., 2015). For instance, the promoting role of miR-99a was illustrated in the radiosensitivity of NSCLC cells (Yin et al., 2018). Song et al. indicated that miR-144-5p radiosensitized the NSCLC cells by targeting ATF2 (Song et al., 2018). Additionally, miR-320a was elucidated to promote cancer cell radiosensitivity (Hu et al., 2018). It has also been well established that miR-320 acts as an anti-oncogene in multiple cancers by inducing apoptosis and inhibiting proliferation, migration, and invasion of cancer cells (Lv G. et al., 2017). Intriguingly, it has been reported that the miR-320a expression was preeminently increased with the increase in irradiation dose and duration of treatment whilst miR-320a promoted the irradiation-induced cell apoptosis in lung adenocarcinoma cells (Lv Q. et al., 2017). Liang et al. (2017) also found that miR-320 inhibited autophagy by targeting hypoxia-inducible factor-1α (HIF1α) in retinoblastoma.

Hypoxia-inducible factor-1α is a critical metabolic sensor in cellular metabolism pathways, and its association with immune responses has been previously demonstrated (Yu et al., 2018). Additionally, it is well-accepted that HIF1α can potentially promote the radioresistance of multiple cancers. For instance, normoxic expression of HIF1α is correlated to the development of radioresistance of prostate cancer cells (Ranasinghe et al., 2015). Another study provided evidence that HIF1α overexpression promotes the radioresistance of NSCLC cells by inducing glycolysis (Jiang et al., 2016). Moreover, NSCLC cells presented with a high expression of HIF1α, which was linked with high proliferation capacity of NSCLC cells (Ding et al., 2013). Peculiarly, HIF1α reportedly activates the expression of histone demethylase KDM5B in liver cancer cells (Xia et al., 2009). However, a recent study demonstrated that patients with lung squamous cell carcinomas were observed to have a high expression of KDM5B but had a poor response to radiation (Bayo et al., 2018), providing a clue that KDM5B could be the factor promoting radiation resistance in lung squamous cell carcinomas. In pancreatic cancer cells, KDM5B could potentially inhibit PTEN expression through its demethylase (H3K4me3) function ([Bibr B45][Bibr B30]). However, another study highlighted the association between activated PTEN and miR-21, which results in inhibition of radioresistance in NSCLC cells (Liu et al., 2013). With these findings taken into consideration, we hypothesized that the implication of miR-320a/HIF1α/KDM5B/PTEN axis in radioresistance of NSCLC. Therefore, NSCLC cells and mice were obtained, transfected, and irradiated to investigate the effect of miR-320a on cancerous cell proliferation and apoptosis following irradiation and the potential involvement of HIF1α, KDM5B, and PTEN was explored.

## Materials and Methods

### Ethics Statement

Patients were informed of study subjects and signed informed consent. This study was approved by the Ethics Committee of Tianjin Medical University Cancer Institute & Hospital. All animal experiments were approved by the Committee on the Ethics of Animal Experiments of Tianjin Medical University Cancer Institute & Hospital and performed in accordance with the National Institutes of Health guide for the care and use of laboratory animals.

### Microarray Analysis

After reviewing the relevant literature, the miRs that affected the radiosensitivity of lung cancer were selected. The miR microarray dataset GSE112374 for lung cancer radiosensitivity were downloaded from the Gene Expression Omnibus (GEO) database^1^. There were eight samples in microarray data, including four irradiated cancer samples and four untreated cancer samples. The differentially expressed miRNAs in microarray data were selected with | log fold change| > 1 and *p-*value < 0.05 used as the screening threshold, followed by analysis. The target genes of miRs were predicted by the Starbase database^2^. Then differential expression analysis of target genes in the TCGA database was performed by the GEPIA online website^3^.

### Sample Collection and Cell Culture

A total of 43 NSCLC patients in Tianjin Medical University Cancer Institute & Hospital, including 30 males and 13 females with the mean age of (61.37 ± 8.12) years were enrolled, with NSCLC and adjacent tissues collected. All enrolled patients received no chemotherapy or radiotherapy prior to surgery. Human NSCLC cell lines (A549, H23, H522, and SPC-A1) and human bronchial epithelial cell (16HBE) purchased from American Type Culture Collection (Manassas, VA, United States) were cultured in a RPMI-1640 complete culture medium (Gibco, Grand Island, NY, United States) containing 10% fetal bovine serum (FBS; Gibco), 100 ug/mL streptomycin, and 100 U/mL penicillin in a 5% CO_2_ incubator at 37°C.

### Cell Transfection

Human NSCLC cell lines (A549 and H23) were respectively transfected with mimic negative control (NC), miR-320a mimic, inhibitor NC, miR-320a inhibitor, mimic NC + overexpressed (oe)-NC, miR-320a mimic + oe-NC, miR-320a mimic + oe-HIF1α, miR-320a mimic + oe-KDM5B, small interfering RNA (si)-NC, si-KDM5B, si-KDM5B + si-PTEN, mimic NC + si-NC, miR-320a mimic + si-NC, and miR-320a mimic + si-PTEN plasmids. The plasmids of oe-NC, oe-HIF1α, and oe-KDM5B were constructed using pcDNA3.1 vectors by Hanbio Biotechnology Co., Ltd. (Shanghai, China), while the plasmids of mimic NC, miR-320a mimic, si-NC, si-KDM5B, and si-PTEN were designed and constructed by Shanghai Gene Pharma Co., Ltd. (Shanghai, China). The A549 and H23 cells were transfected with the aforementioned plasmids using Lipofectamine^TM^ 2000 (Invitrogen, Carlsbad, CA, United States) according to the manufacturer’s instructions. The concentration of the transfected plasmids was 100 μM, and the cells were collected after 24 h of transfection for subsequent experiments. Next, in order to culture the cells under hypoxic condition, 5 h post-successful transfection, the A549 and H23 cells were cultured at 37°C in an incubator containing 1% O_2_, 5% CO^2^, and 94% N^2^ for 24 h in order to culture the cells under hypoxic condition (Yang et al., 2018). Cells were collected 24 h post-transfection for subsequent use.

### Radiation Treatment

The cells and mice were treated with radiation using an X-ray machine (X-RAD 320, Precision X-ray Inc., North Branford, CT, United States). For cells, the transfected A549 and H23 cells were treated at a dose rate of 2 Gy/min at room temperature to achieve the required total dose. For mice, the detail radiation procedures were described previously in the “Xenograft Tumor in Nude Mice” methods (Chen et al., 2011; Lan et al., 2015; Kwon et al., 2016; Fan et al., 2020).

### RNA Isolation and Quantitation

Total RNA was extracted using an RNeasy Mini Kit (Qiagen, Valencia, CA, United States). Then, according to the instructions, the mRNA was reversely transcribed into complementary DNA (cDNA) with the use of PrimeScript RT kit (RR047A, Takara, Tokyo, Japan), while the miR using a miRNA First Strand cDNA Synthesis (Tailing Reaction) kit (B532451-0020, Sangon Biotech Co., Ltd., Shanghai, China). Subsequently, fluorescence quantitative PCR was carried out in a SYBR^®^ Premix Ex Taq^TM^ II kit (DRR081, Takara) on ABI 7500 quantitative PCR appliance (Thermo Fisher Scientific, Foster City, CA, United States). The miRNA universal reverse primer and the upstream primer of U6 were provided by the miRNA First Strand cDNA Synthesis (Tailing Reaction) kit while the remaining primers were synthesized by Sangon Biotech Co., Ltd. The detail primer sequences are depicted in Table 1. The relative transcription levels were analyzed using the 2^–ΔΔCt^ method with glyceraldehyde-3-phosphate dehydrogenase (GAPDH) or U6 used as internal references (Ayuk et al., 2016).

**TABLE 1:** Primer sequences for RT-qPCR.

**Gene**	**Primer sequence**
miR-320a	F:5′-AAAAGCTGGGTTGAGAGGGCGA-3′
HIF1α	F: 5′-GAACGTCGAAAAGAAAAGTCTCG-3′
	R: 5′-CCTTATCAAGATGCGAACTCACA-3′
KDM5B	F: 5′-CCATAGCCGAGCAGACTGG-3′
	R: 5′-GGATACGTGGCGTAAAATGAAGT-3′
PTEN	F: 5′-TTTGAAGACCATAACCCACCAC-3′
	R: 5′-ATTACACCAGTTCGTCCCTTTC-3′
GAPDH	F: 5′-GGAGCGAGATCCCTCCAAAAT-3′
	R: 5′-GGCTGTTGTCATACTTCTCATGG-3′

*RT-qPCR, reverse transcription quantitative polymerase chain reaction; miR-320a, microRNA-320a; HIF1α, hypoxia-inducible factor 1α; KDM5B, lysine demethylase 5B; PTEN, phosphatase and tensin homolog; F, forward; R, reverse.*

### Western Blot Analysis

Total protein was extracted using radio-immune precipitation assay lysis buffer containing phenylmethylsulfonyl. Subsequently, 50 μg of protein was subjected to sodium dodecyl sulfate polyacrylamide gel electrophoresis, after which it was transferred to a polyvinylidene fluoride membrane. The membrane was subsequently blocked by 5% skimmed milk for 1 h and incubated with the diluted mouse anti-rabbit primary antibodies (Abcam, Cambridge, United Kingdom) to HIF1α (ab216842, 1:500), KDM5B (ab181089, 1:1000), PTEN (ab170941, 1:1000), Ki67 (ab92742,1:5000), Bax (ab32503, 1:1000), and GAPDH (ab9485, 1:2500, as internal control) overnight at 4°C. The membrane underwent incubation with horseradish peroxidase-labeled goat anti-rabbit IgG secondary antibody (ab97051, 1:2000, Abcam) for 1 h. The membrane was immersed in enhanced chemiluminescence solution (BB-3501, Amersham Pharmacia Biotech, Piscataway, NJ, United Kingdom). Afterward, the membrane was observed in a darkroom and photographed using a Bio-Rad image analysis system (Bio-Rad, Hercules, CA, United States). Quantity One v4.6.2 software was used for density analysis of bands and the ratio of the gray value of the target band to the internal reference band was regarded as the relative expression of the protein.

### Dual-Luciferase Reporter Gene Assay

The wild type (WT) and mutant type (MUT) reporter plasmids of HIF1α-3′UTR (pGL3-WT-HIF1α-3′UTR and pGL3-MUT-HIF1α-3′UTR) were designed by Shanghai Gene Pharma Co., Ltd. The detailed procedure was as follows (Zhong et al., 2019): According to the binding sequence of HIF1α mRNA 3′UTR region with miR-30a, the target sequence and mutation sequence were designed, after which the target sequence was chemically synthesized. The sequence was added with *Xho*I and *Not*I digestion sites at both ends during synthesis. The synthesized fragments were cloned into the PUC57 vector. After the identification of positive clones, the recombinant plasmids were identified by DNA sequencing, then sub-cloned into the pGL3-basic vector and transformed into Escherichia coli DH5α cells to amplify the plasmids. All of the aforementioned plasmids were extracted according to the manuals of Omega Plasmid Small Quantity Extraction Kit (D1100-50T, Beijing Solabio Life Sciences Co., Ltd., Beijing, China). The mimic NC and miR-320a mimic were co-transfected with WT-HIF1α-3′UTR and MUT-HIF1α-3′UTR, respectively, into A549 or H23 cells. The proportion of plasmid transfection was identified by the firefly luciferase reporter gene vector: mimic: pRL-TK = 1 μg: 100 nM: 0.2 μg. After 48 h of transfection, the cells were lysed, and the luciferase reporter gene assay was performed according to the instructions of luciferase assay kit (K801-200, Biovision, Mountain View, CA, United States) using a dual-luciferase reporter assay system (Promega, Madison, WI, United States). Each cell sample was added with 100 μL firefly luciferase working fluid to detect firefly luciferase and with 100 μL of Renilla luciferase working fluid to detect Renilla luciferase. Luciferase activity of activation of the target reporter gene was regarded as the ratio of the firefly luciferase assay value to the Renilla luciferase assay value, with pRL-TK Renilla luciferase plasmid used as an internal reference. The profiles of the firefly luciferase reporter plasmid vector and the Renilla luciferase reporter plasmid vectors are shown in Supplementary Figure S1.

### Chromatin Immunoprecipitation (ChIP)

Chromatin immunoprecipitation experiments were performed using an EZ-Magna ChIP A/G Chromatin Immunoprecipitation Kit (17–371, Millipore, Billerica, MA, United States) according to the manufacturer’s instructions. After sonication, the cells were centrifuged at 12000 × *g* for 10 minutes at 4°C for the removal of the insoluble precipitate. Then, cells were incubated with Protein G Agarose at 4°C for 1 h and centrifuged at 5000 × *g* for 1 min. After that, 10 μL (1%) supernatant was taken as “Input control.” The remaining supernatant was divided into two parts, which were added with H3K4me3 antibody (9751S, 1:50, Cell Signaling Technology, Massachusetts, MA, United States) and NC rabbit anti-human IgG (ab2410, 1:25, Abcam) respectively, followed by overnight incubation at 4°C for full binding. The protein and DNA complexes were precipitated by protein G Agarose, followed by incubation at 4°C for 1 h. After centrifugation at 5000 × *g* for 1 minute, the supernatant was discarded, and the protein and DNA complexes were eluted. After de-crosslinking, overnight at 65°C, the DNA fragments were purified and recovered. RT-qPCR experiment was carried out by recovering the purified DNA fragment as an amplification template.

### Cell Apoptosis Detection

The cells were treated with 10 Gy X-rays at 24 h after transfection and cultured for another 24 h. Then the cell apoptosis was measured with annexin V-fluorescein isothiocyanate (FITC)/PI Kit, according to the manufacturer’s instructions (KeyGEN Biotechnology Co., Ltd., Nanjing, China). The results were analyzed using a flow cytometer (FACSCalibur, BD Biosciences).

### Clonogenic Survival Analysis

A total of 300 viable cells were seeded in 6-cm-thick dishes and cultured using fresh complete medium. After the cells adhered, the cells were treated using a radiation dose of 10 Gy, and then cultured for 10–12 d. After visible colony formation, the cells were fixed with 4% paraformaldehyde and stained with crystal violet (Solarbio, Beijing, China). Colonies with ≥ 50 cells were counted under a microscope and images were captured with a camera. The survival fraction (SF) was calculated as follows: SF = number of colonies formed/number of cells seeded × 100%. The experiment was repeated three times.

### Xenograft Tumor in Nude Mice

Thirty male BALB/C nude mice (6–8 weeks old, weight 15–18 g) were selected from Beijing Vital River Laboratory Animal Technology Co., Ltd. (Beijing, China) and housed in pathogen-free animal facilities. They were randomly grouped into three groups by respective treatment with lentivirus vectors (Lv)-oe-NC + Lv-sh-NC, Lv-oe-miR-320a + Lv-sh-NC, and Lv-oe-miR-320a + Lv-sh-PTEN (*n* = 10 in each group). Lentiviral vectors Lv-oe-NC, Lv-oe-miR-320a, Lv-sh-NC and Lv-sh-PTEN were purchased from Shanghai Gene Pharma Co., Ltd and constructed according to the following method (Li et al., 2019): recombinant lentiviral expression vectors (Lv-oe-miR-320a and Lv-sh-PTEN) with green fluorescence protein gene were constructed. To generate lentiviral particles, the recombinant expression plasmids were co-transfected with a packaging plasmid system (psPAX2 and pMD2G) into HEK-293T cells and viral particles were collected after 48 h of transfection. The A549 cells were then infected with the indicated lentiviral vector for 24 h. The infection efficiency was preliminarily assessed in each experiment under a fluorescence microscope and then measured by sorting the positive cells of green fluorescence using flow cytometry (Beckman Coulter, Brea, CA, United States). The miR stably expressed cells were amplified and harvested for further experiments. The mice were initially anesthetized with a mixture of zoletil (30 mg/kg) and rompun (10 mg/kg). After the lentiviral infection, the stably transfected A549 cells (5 × 10^6^) were selected and subcutaneously injected into the right abdomen of nude mice. Three days later, the mice received local treatment with 10 Gy X-rays every 3 days for a total of 10 times. After 30 days of injection, the nude mice were euthanized by carbon dioxide asphyxiation and the tumors were weighed, followed by subsequent experiments. The volume of the tumor was measured every 3 days as follows: tumor volumes (mm^3^) = a × b^2^/2, where a and b were the maximum and minimum tumor diameters, respectively. The growth curve was drawn.

### Statistical Analysis

Data were analyzed using SPSS 21.0 software (IBM Corp. Armonk, NY, United States). The measurement data were presented as mean ± standard deviation. The Shapiro-Wilk test was used to test normality. If conforming to normal distribution and homogeneity of variance, the paired *t*-test was used to compare the paired data between the two groups and the unpaired *t*-test was used to compare the unpaired data between the two groups. A comparison for more than two groups was analyzed by one-way analysis of variance (ANOVA) with Tukey’s *post hoc* test. Data comparison between groups at different time points was performed by repeated-measures ANOVA and Bonferroni was performed for *post hoc* test. The correlation analysis was performed by Pearson’s correlation coefficient. When the *p*-value was less than 0.05, it was considered to be statistically significant.

## Results

### miR-320a Is Lowly-Expressed in NSCLC Tissues and Cells and Correlates to Radiosensitivity of NSCLC

**FIGURE 1:**
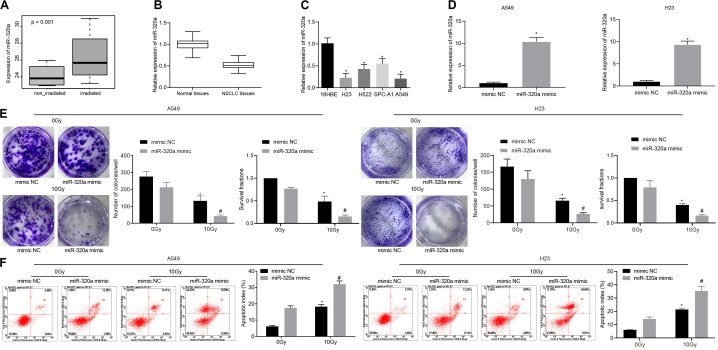
miR-320a promoted the radiosensitivity of NSCLC. **(A)** The differential analysis of human NSCLC radiosensitivity microarray data GSE112374 in the GEO database. **(B)** Quantitative analysis of the expression of miR-320a in human NSCLC tissues and adjacent tissues. *p < 0.05 compared with adjacent tissue. **(C)** Quantitative analysis of the expression of miR-320a in NSCLC cell lines (A549, H23, H522, and SPC-A1) and human bronchial epithelial cells (16HBE). *p < 0.05 compared with 16HBE cell. **(D)** Quantitative analysis of the transfection efficiency of miR-320a in A549 and H23 cells. *p < 0.05 compared with cells treated with mimic NC. **(E)** Clonogenic survival analysis of the number of colony formation and SFs in A549 and H23 cells at 24 h after irradiation treatment. **(F)** Flow cytometry detection of the apoptosis index in A549 and H23 cells at 24 h after irradiation treatment (Apoptotic rate = sum of Q2 + Q4 data). *p < 0.05 compared with cells after 0 Gy X-ray treatment. ^#^*p* < 0.05 compared with cells after 10 Gy X-ray treatment. The results were measurement data, which were expressed as the mean ± standard deviation *n* = 43. Comparisons between two groups were conducted using paired *t*-test **(B)**, and comparisons between multiple groups analyzed by one-way ANOVA **(C)** with Tukey’s *post hoc* test. Comparisons between the two groups were conducted using an unpaired *t*-test **(D–F)**. The experiment was independently repeated three times.

The differential analysis of human NSCLC radiosensitivity microarray dataset GSE112374 in the GEO database was performed (Figure 1A) and our results revealed the high expression of miR-320a in radiosensitivity samples of NSCLC. The data from RT-qPCR experiment exhibited the significant down-regulation of miR-320a in NSCLC tissues (*n* = 43) and in 4 NSCLC cell lines (A549, H23, H522, and SPC-A1) as compared to the corresponding adjacent tissues (*n* = 43) and the human bronchial epithelial cell (16HBE), respectively (Figures 1B,C). Therefore, A549 and H23 cell lines with relatively low expression of miR-320a were selected for subsequent cell experiments. Moreover, A549 and H23 cells were transfected with mimic NC and miR-320a mimic for 24 h followed by exposure to a total dose of 10 Gy X-ray with a dose rate of 2 Gy/min and further 24-hour culture. Intriguingly, RT-qPCR revealed that the resultant transfection efficiency of miR-320a met the standard for further experiments (*p* < 0.05; Figure 1D). Thereafter, the number of colony formation and SFs of each group of cells was detected with the application of clonogenic survival analysis (Figure 1E). Our results demonstrated that after 0 Gy X-ray treatment, the number of colony formation and SFs of cells treated with miR-320a mimic was significantly lower than that of cells treated with mimic NC (*p* < 0.05) whereas following exposure to 10 Gy X-ray, the number of colony formation and SFs of cells was significantly reduced by treatment with miR-320a mimic (*p* < 0.05), indicating that miR-320a could potentially promote the radiosensitivity of NSCLC. Moreover, our flow cytometry results (Figure 1F) indicated that cells treated with miR-320a mimic + 0 Gy or mimic NC + 10 Gy markedly promoted apoptosis index of cells relative to those treated with mimic NC + 0 Gy. Under the treatment of 10 Gy X-ray, miR-320a mimic treatment also enhanced apoptosis index of cells in comparison with mimic NC treatment. The results supported the hypothesis that miR-320a could promote radiosensitivity of NSCLC.

### miR-320a Suppresses the Radioresistance of NSCLC Through Inhibition of HIF1α

**FIGURE 2:**
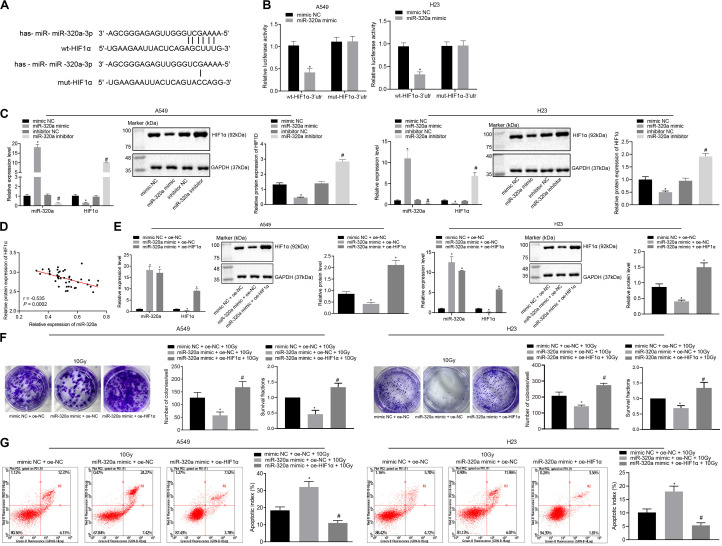
miR-320a suppressed the radioresistance of NSCLC through inhibition of HIF1α. **(A)** Microarray analysis of the relationship between HIF1α and miR-320a. **(B)** Dual-luciferase reporter gene assay for verifying the relationship between HIF1α and miR-320a. *p < 0.05 compared with cells stimulated with mimic NC. **(C)** The effect of miR-320a on the expression of HIF1α in A549 and H23 cells was measured by RT-qPCR and western blot analysis. *p < 0.05 compared with cells stimulated with mimic NC. **(D)** Pearson correlation analysis of the expression of miR-320a and HIF1α in 43 cases of NSCLC. **(E)** The transfection efficiency of A549 and H23 cells detected by RT-qPCR and western blot analysis. *p < 0.05 compared with cells stimulated with mimic NC. **(F)** The number of colony formation and SFs of A549 and H23 cells detected by clonogenic survival analysis at 24 h after irradiation treatment. **(G)** The apoptosis index of A549 and H23 cells detected by flow cytometry at 24 h after irradiation treatment (Apoptotic rate = sum of Q2 + Q4 data). *p < 0.05 compared with cells stimulated with mimic NC + oe-NC + 10 Gy. ^#^*p* < 0.05 compared with cells stimulated with miR-320a mimic + oe-NC + 10 Gy. The results were measurement data, which were expressed as the mean ± standard deviation *n* = 43. Comparisons between the two groups were conducted using an unpaired *t*-test **(B,C)** and comparisons between multiple groups analyzed by one-way ANOVA **(E–G)** with Tukey’s *post hoc* test. The experiment was independently repeated three times.

In order to explore the relationship between HIF1α and miR-320a, microarray analysis, dual-luciferase reporter gene assay, RT-qPCR, western blot analysis, and Pearson correlation analysis were carried out. Initially, the target regulatory gene of miR-320a was predicted from the Starbase database, and we found that miR-320a had a binding site with transcription factor HIF1α (UCGAAAA) (Figure 2A). According to dual-luciferase reporter gene assay (Figure 2B), co-transfection of miR-320a mimic with WT-HIF1α-3′UTR resulted in a significant decrease in luciferase activity compared with co-transfection with mimic NC and WT-HIF1α-3′UTR (*p* < 0.05), while there was no significant difference observed in luciferase activity between cells co-transfected with miR-320a mimic and MUT-HIF1α-3′UTR and cells co-transfected with mimic NC and MUT-HIF1α-3′UTR (*p* > 0.05). After cells were treated with inhibitor NC, miR-320a inhibitor, NC mimic, or miR-320a mimic, the results of RT-qPCR and western blot analysis depicted that miR-320a negatively regulated the expression of HIF1α in A549 and H23 cells (Figure 2C). Moreover, Pearson correlation analysis was also confirmed the negative relation between miR-320a and HIF1α in NSCLC tissues (Figure 2D). Thereafter, to explore the effect of miR-320a on radioresistance of NSCLC via HIF1α, A549 and H23 cells were stimulated with mimic NC + oe-NC + 10 Gy, miR-320a mimic + oe-NC + 10 Gy, and miR-320a mimic + oe-HIF1α + 10 Gy. Our results from RT-qPCR showed that the transfection efficiency of A549 and H23 cells achieved the requirements for further experiments (*p* < 0.05; Figure 2E). Subsequently, the number of colony formation and SFs of A549 and H23 cells was detected by clonogenic survival analysis. As documented in Figure 2F, the number of colony formation and SFs of cells stimulated with miR-320a mimic + oe-NC + 10 Gy was significantly lower than that of cells stimulated with mimic NC + oe-NC + 10 Gy; compared with cells stimulated with miR-320a mimic + oe-NC + 10 Gy, the number of colony formation and SFs of cells stimulated with miR-320a mimic + oe-HIF1α + 10Gy cells was notably increased (all *p* < 0.05). Finally, in contrast to cells stimulated with mimic NC + oe-NC + 10 Gy, the apoptosis index of cells stimulated with miR-320a mimic + oe-NC + 10Gy was significantly increased (*p* < 0.05). In the presence of miR-320a mimic + 10 Gy, HIF1α overexpression significantly reduced the apoptosis index (*p* < 0.05; Figure 2G). Collectively, the above data suggested that miR-320a could inhibit the radioresistance of NSCLC through its inhibitory role on HIF1α.

### miR-320a Inhibits HIF1α and KDM5B (JARID1B), Thereby Inhibiting the Radioresistance of NSCLC

**FIGURE 3:**
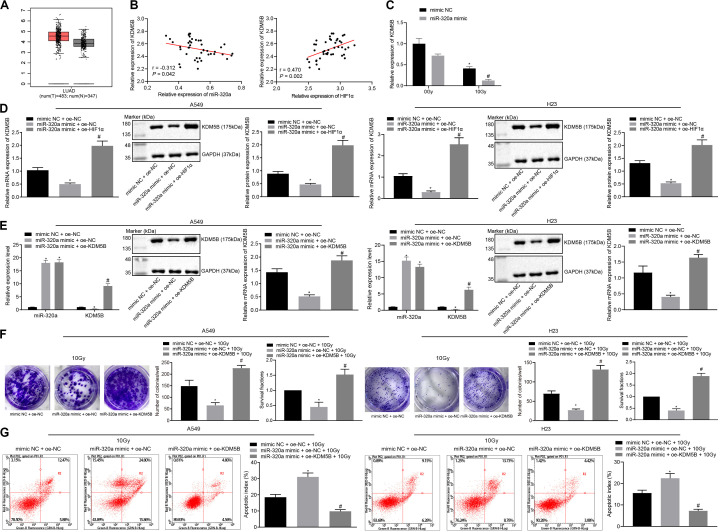
miR-320a suppressed the radioresistance of NSCLC by down-regulation of HIF1α and KDM5B. **(A)** The differential expression of histone demethylase KDM5B in all cancers in the TCGA database. **(B)** Pearson correlation analysis of the expression of miR-320a and HIF1α as well as HIF1α and KDM5B in 43 cases of NSCLC. **(C)** The expression of KDM5B in A549 and H23 cells measured by RT-qPCR *p < 0.05 compared with cells stimulated with mimic NC + 0 Gy. ^#^*p* < 0.05 compared with cells stimulated with miR-320a mimic + 0 Gy. **(D)** The effect of a miR-320a-HIF1α axis on the expression of KDM5B in A549 and H23 cells measured by RT-qPCR and western blot analysis. *p < 0.05 compared with cells stimulated with mimic NC + oe-NC. ^#^*p* < 0.05 compared with cells stimulated with miR-320a mimic + oe-NC. **(E)** The transfection efficiency in A549 and H23 cells detected by RT-qPCR and western blot analysis. *p < 0.05 compared with cells stimulated with mimic NC + oe-NC. ^#^*p* < 0.05 compared with cells stimulated with miR-320a mimic + oe-NC. **(F)** The number of colony formation and SFs of A549 and H23 cells detected by clonogenic survival analysis at 24 h after irradiation treatment. *p < 0.05 compared with cells stimulated with mimic NC + oe-NC + 10Gy. ^#^*p* < 0.05 compared with cells stimulated with miR-320a mimic + oe-NC + 10Gy. **(G)** The apoptosis index of A549 and H23 cells were detected by flow cytometry at 24 h after irradiation treatment (Apoptotic rate = sum of Q2 + Q4 data). *p < 0.05 compared with cells stimulated with mimic NC + oe-NC + 10 Gy. ^#^*p* < 0.05 compared with cells stimulated with miR-320a mimic + oe-NC + 10Gy. The results were measurement data, which were expressed as the mean ± standard deviation. *n* = 43. Comparisons between the two groups were conducted using unpaired *t*-test. Comparisons between multiple groups were analyzed by one-way ANOVA with Tukey’s *post hoc* test. The experiment was independently repeated three times.

The underlying mechanism of miR-320a and HIF1α in NSCLC has been previously reported and provided evidence that HIF1α could induce the KDM5B expression (Salminen et al., 2016). Therefore, here we speculated that miR-320a could inhibit the expression of HIF1 α and KDM5B, thus repressing the radioresistance of NSCLC. To test this hypothesis, we first analyzed the differential expression of histone demethylase KDM5B in all cancers in The Cancer Genome Atlas (TCGA) database through Gene Expression Profiling Interactive Analysis (GEPIA) online website (see text Footnote 3). It was found that KDM5B was highly expressed in lung adenocarcinoma (Figure 3A). Furthermore, Pearson correlation analysis showed that the expression of miR-320a and KDM5B was negatively correlated in NSCLC tissues while the expression of HIF1α and KDM5B was positively correlated (Figure 3B). To investigate the impact of KDM5B on the miR-320a-dependent radiosensitization, RT-qPCR was performed to detect the KDM5B expression. As depicted in Figure 3C, KDM5B expression was markedly reduced in the cells treated with miR-320a mimic + 0 Gy or mimic NC + 10 Gy compared with those that received treatment with mimic NC + 0 Gy. Under 10 Gy X-ray, miR-320a mimic treatment also induced reduced KDM5B expression relative to mimic NC treatment. Thereafter, to investigate the effect of the miR-320a-HIF1α axis on KDM5B expression, miR-320a and HIF1α were overexpressed in A549 and H23 cells followed by detection of the expression of KDM5B in cells using RT-qPCR and western blot analysis (Figure 3D). Our results showed that the expression of KDM5B was significantly reduced secondary to treatment with miR-320a mimic, but it was reversed by additional treatment with oe-HIF1α (all *p* < 0.05). Thereafter, to explore the effect of miR-320a on the radioresistance of NSCLC through regulation of KDM5B by targeting HIF1α, A549 and H23 cells were treated with mimic NC + oe-NC + 10 Gy, miR-320a mimic + oe-NC + 10 Gy, and miR-320a mimic + oe-KDM5B + 10 Gy. RT-qPCR and western blot analysis findings suggested that the transfection efficiency of A549 and H23 cells had reached the requirements for subsequent experiments (*p* < 0.05; Figure 3E). Then the number of colony formation and SFs of A549 and H23 cells was detected with the use of clonogenic survival analysis (Figure 3F). In the presence of 10 Gy X-ray, the number of colony formation and SFs of cells after miR-320a mimic treatment was significantly lowered, which was abrogated following treatment with oe-HIF1α (all *p* < 0.05). Furthermore, the results of flow cytometry (Figure 3G) revealed that the apoptosis index of cells stimulated with miR-320a mimic + oe-NC + 10 Gy was notably enhanced compared with cells stimulated with mimic NC + oe-NC + 10 Gy (*p* < 0.05). Compared with cells stimulated with miR-320a mimic + oe-NC + 10 Gy, the apoptosis index of miR-320a mimic + oe-HIF1α + 10 Gy was significantly diminished (*p* < 0.05). Taken together, miR-320a was found to suppress HIF1α and KDM5B, thereby inhibiting the radioresistance of NSCLC.

### KDM5B Promoted the Radioresistance of NSCLC via Inhibition of PTEN

**FIGURE 4:**
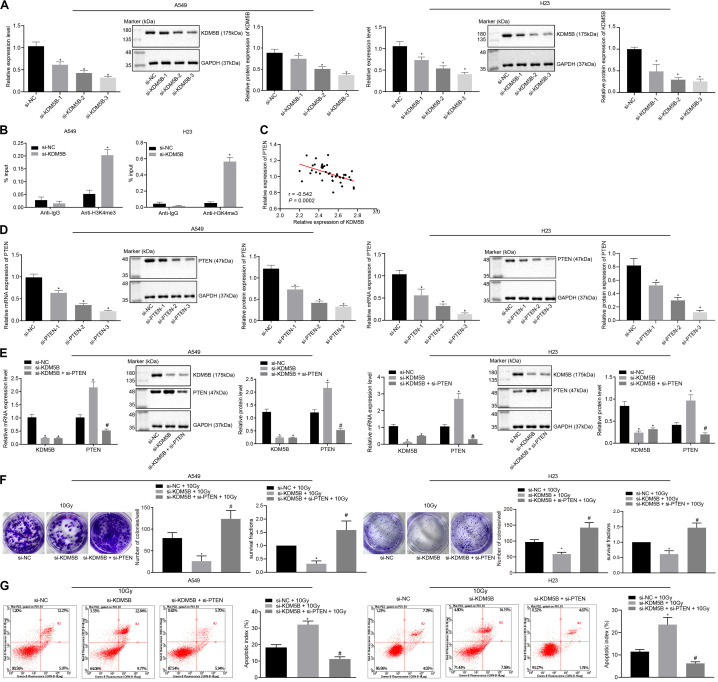
KDM5B enhanced the radioresistance of NSCLC through suppression of PTEN. **(A)** The silencing efficiency of KDM5B in A549 and H23 cells measured by RT-qPCR and western blot analysis (Cells were collected 24 h after siRNA transfection). *p < 0.05 compared with cells stimulated with si-NC. **(B)** The content of PTEN promoter fragment enriched by IgG antibody and H3K4me3 antibody in A549 and H23 cells measured using ChIP assay. *p < 0.05 compared with cells stimulated with anti-IgG. **(C)** Pearson correlation analysis of the expression of KDM5B and PTEN in 43 cases of NSCLC. **(D)** The silencing efficiency of PTEN in A549 and H23 cells measured by RT-qPCR and western blot analysis. *p < 0.05 compared with cells stimulated with si-NC. **(E)** The transfection efficiency of PTEN and KDM5B in A549 and H23 cells detected by RT-qPCR and western blot analysis. *p < 0.05 compared with cells stimulated with si-NC. ^#^*p* < 0.05 compared with cells stimulated with si-KDM5B. **(F)** The number of colony formation and SFs of A549 and H23 cells detected by clonogenic survival analysis at 24 h after irradiation treatment. *p < 0.05 compared with cells stimulated with si-NC + 10 Gy. ^#^*p* < 0.05 compared with cells stimulated with si-KDM5B + 10 Gy. **(G)** The apoptosis index of A549 and H23 cells detected by flow cytometry at 24 h after irradiation treatment (Apoptotic rate = sum of Q2 + Q4 data). *p < 0.05 compared with cells stimulated with si-NC + 10 Gy. ^#^*p* < 0.05 compared with cells stimulated with si-KDM5B + 10 Gy. The results were measurement data, which were expressed as the mean ± standard deviation *n* = 43. Comparisons between multiple groups were analyzed by one-way ANOVA **(A,D)** with Tukey’s *post hoc* test. Comparisons between the two groups were conducted using an unpaired *t*-test **(B,E–G)**.

To investigate the role of KDM5B and PTEN in the radioresistance of NSCLC, small interfering (si)-KDM5B-3 i.e., [the best silencing efficiency of the three small interfering RNAs (siRNAs) for silencing KDM5B] were selected for subsequent experiments (Figure 4A). Then, KDM5B in A549 and H23 cells were silenced and ChIP assay was performed to detect the content of PTEN promoter fragment enriched by Immunoglobulin G (IgG) antibody and H3K4me3 antibody (Figure 4B). ChIP assay showed that H3K4me3 enrichment in A549 and H23 cells were significantly increased in the PTEN promoter region after silencing KDM5B, suggesting that KDM5B could also demethylate H3K4me3 in the PTEN promoter region in NSCLC. Moreover, we also found that there was a negative correlation between the expression of KDM5B and PTEN in NSCLC, as indicated by the results obtained from Pearson correlation analysis (Figure 4C). Afterward, A549 and H23 cells were treated with si-NC + 10 Gy, si-KDM5B + 10 Gy, and si-KDM5B + si-PTEN + 10 Gy. For the siRNA used to silence PTEN, si-PTEN-3 with the best silencing efficiency was selected (Figure 4D). RT-qPCR and Western blot analysis demonstrated that the transfection efficiency of cells had reached the requirements of further experiments (*p* < 0.05; Figure 4E). Moreover, these results also showed that the expression of PTEN could be up-regulated following the silencing of KDM5B. Consistent with our mechanism study of Figure 4B, KDM5B demethylated the H3K4me3 in the PTEN promoter region in NSCLC cells to inhibit PTEN expression. Afterward, as depicted in Figure 4F, in the presence of 10 Gy X-ray, the number of colony formation and SFs of cells was reduced by silencing KDM5B, which was neutralized by silencing PTEN (all *p* < 0.05). Lastly, the results from flow cytometry (Figure 4G) manifested that compared with cells stimulated by si-NC + 10 Gy, the apoptosis index of cells stimulated with si-KDM5B + 10 Gy was notably increased (*p* < 0.05). Whilst compared with cells stimulated with si-KDM5B + 10 Gy, the apoptosis index of si-KDM5B + si-PTEN + 10 Gy was significantly diminished (*p* < 0.05). These findings suggested that KDM5B could enhance the radioresistance of NSCLC via inhibition of PTEN.

### miR-320a/HIF1α/KDM5B/PTEN Axis Is Involved in the Radioresistance of NSCLC

**FIGURE 5:**
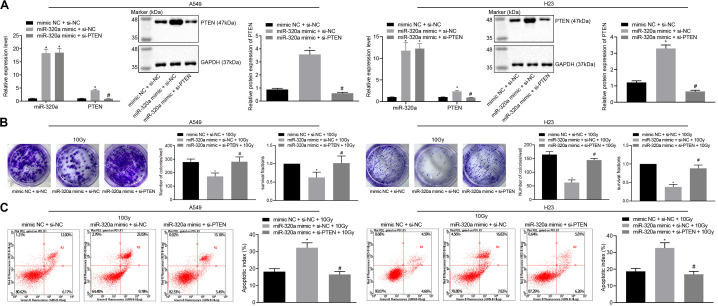
miR-320a/HIF1α/KDM5B/PTEN axis involved in the radioresistance of NSCLC cells. **(A)** The transfection efficiency in A549 and H23 cells detected by RT-qPCR and western blot analysis. *p < 0.05 compared with cells stimulated with mimic NC + si-NC. ^#^*p* < 0.05 compared to cells stimulated with miR-320a mimic + si-NC. **(B)** The number of colony formation and SFs of A549 and H23 cells detected by clonogenic survival analysis at 24 h after irradiation treatment. *p < 0.05 compared with cells stimulated with mimic NC + si-NC + 10 Gy. ^#^*p* < 0.05 compared with cells stimulated with miR-320a mimic + si-NC + 10 Gy. **(C)** The apoptosis index of A549 and H23 cells detected by flow cytometry at 24 h after irradiation treatment (Apoptotic rate = sum of Q2 + Q4 data). *p < 0.05 compared with cells stimulated with mimic NC + si-NC + 10 Gy. ^#^*p* < 0.05 compared with cells stimulated with miR-320a mimic + si-NC + 10 Gy. The results were measurement data, which were expressed as the mean ± standard deviation *n* = 43. Comparisons between the two groups were conducted using an unpaired *t*-test. Comparisons between multiple groups were analyzed by one-way ANOVA with Tukey’s *post hoc* test. The experiment was independently repeated three times.

To further measure the regulation of miR-320a/HIF1α/KDM5B/PTEN axis in radioresistance of NSCLC, A549, and H23 cells were stimulated with mimic NC + si-NC + 10 Gy, miR-320a mimic + si-NC + 10 Gy, and miR-320a mimic + si-PTEN + 10 Gy. The transfection efficiency in cells was confirmed by RT-qPCR and western blot analysis (*p* < 0.05; Figure 5A). Besides, the number of colony formation and SFs of A549 and H23 cells was detected by clonogenic survival analysis (Figure 5B). Our results indicated that the number of colony formation and SFs of cells stimulated with miR-320a mimic + si-NC + 10 Gy was notably lower than that of cells stimulated with mimic NC + si-NC + 10 Gy; compared with cells stimulated with miR-320a mimic + si-NC + 10 Gy, the number of colony formation and SFs of cells stimulated with miR-320a mimic + si-PTEN + 10 Gy cells was significantly increased (all *p* < 0.05). Lastly, the results from flow cytometry (Figure 5C) demonstrated that compared with cells stimulated with mimic NC + si-NC + 10 Gy, the apoptosis index of cells stimulated with miR-320a mimic + si-NC + 10 Gy was significantly enhanced (*p* < 0.05). In comparison with cells stimulated with miR-320a mimic + si-NC + 10 Gy, the apoptosis index of miR-320a mimic + si-PTEN + 10 Gy was notably declined (*p* < 0.05). These results collectively demonstrated that in NSCLC cells, miR-320a could inhibit the radioresistance of NSCLC by promoting the expression of PTEN.

### miR-320a Suppressed the Radioresistance of NSCLC in Mouse Xenografts

**FIGURE 6:**
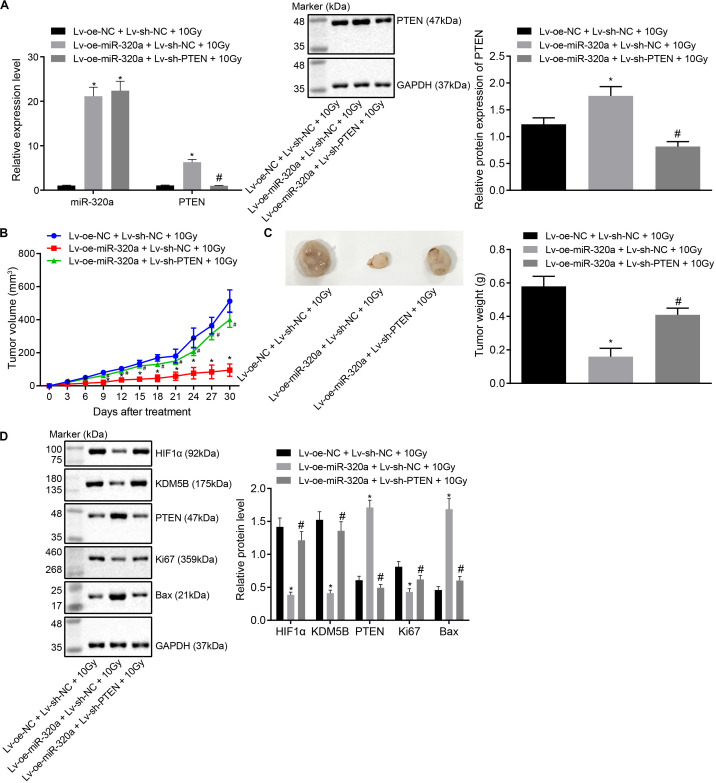
miR-320a inhibited the radioresistance of NSCLC in mouse xenografts. **(A)** The reliability of the stably infected cells confirmed by RT-qPCR and western blot analysis. **(B)** The changes in the volume of xenografts after injection. **(C)** The changes in weight of xenografts after injection. **(D)** Western blot analysis of the expression of HIF1α, KDM5B, PTEN, Ki67 and Bax in the nude mice xenografts. *p < 0.05 compared with mice treated with Lv-oe-NC + Lv-sh-NC + 10 Gy. ^#^*p* < 0.05 compared with mice treated with Lv-oe-miR-320a + Lv-sh-PTEN + 10 Gy. Mice were treated with Lv-oe-miR-320a + Lv-sh-NC + 10 Gy, Lv-oe-miR-320a + Lv-sh-PTEN + 10 Gy, and Lv-oe-NC + Lv-sh-NC + 10 Gy. The results were measurement data, which were expressed as the mean ± standard deviation *n* = 10. Comparisons between the two groups were conducted using an unpaired *t*-test. Comparisons between multiple groups were analyzed by one-way ANOVA with Tukey’s *post hoc* test. Repeated-measures analysis of variance was used for data comparison between groups at different time points and Bonferroni was performed for *post hoc* test.

Thereafter, to further elucidate the effect of miR-320a/HIF1α/KDM5B/PTEN axis on radioresistance of NSCLC xenografts *in vivo*, BALB/c nude mice were treated with lentiviral vector (Lv)-oe-NC + Lv-sh-NC + 10 Gy, Lv-oe-miR-320a + Lv-sh-NC + 10 Gy, and Lv-oe-miR-320a + Lv-sh-PTEN + 10 Gy. Prior to animal experiments, the stably infected A549 cells were confirmed with the application of RT-qPCR and western blot analysis (Figure 6A). After animal experiments, the volume and weight of the nude mice xenografts were recorded, the results of which documented that mice treated with Lv-oe-miR-320a + Lv-sh-NC + 10 Gy had significantly increased the volume and weight of nude mice xenografts (all *p* < 0.05). Compared with mice treated with Lv-oe-miR-320a + Lv-sh-NC + 10 Gy, the volume and weight of nude mice xenografts were significantly decreased in mice treated with Lv-oe-miR-320a + Lv-sh-PTEN + 10 Gy (all *p* < 0.05; Figures 6B,C). Finally, the expression of HIF1α, KDM5B, PTEN, Ki67, and Bcl-2-associated X (Bax) was determined in tissues with the use of western blot analysis (Figure 6D). Intriguingly, our results showed that compared with mice treated with Lv-oe-NC + Lv-sh-NC + 10 Gy, mice treated with Lv-oe-miR-320a + Lv-sh-NC + 10 Gy had significantly reduced the expression of HIF1α, KDM5B and Ki6, but the expression of PTEN and Bax was enhanced (all *p* < 0.05). Moreover, compared with mice treated with Lv-oe-miR-320a + Lv-sh-NC + 10 Gy, mice treated with Lv-oe-miR-320a + Lv-sh-PTEN + 10 Gy showed significantly increased expression of HIF1α, KDM5B, and Ki67 while PTEN and Bax expression was decreased (all *p* < 0.05). All data suggested that miR-320a could suppress the radioresistance of NSCLC in mouse xenografts.

## Discussion

**FIGURE 7:**
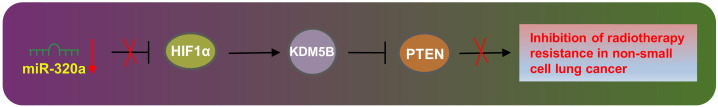
The underlying mechanism concerning miR-320a in NSCLC was explored. Elevated miR-320a inhibited expression of HIF1α and KDM5B. Besides, KDM5B could enhance the radioresistance of NSCLC via inhibition of PTEN. Further, up-regulated miR-320a inhibited radioresistance of NSCLC by promoting methylation of PTEN through repressing HIF1α.

Non-small cell lung cancer is a malignant tumor that accounts for 80% of lung cancers, the diagnosis of which is mostly made in late stages, resulting in a low number of colony formation and SFs (Digesu et al., 2018). Moreover, the emergence of chemo- or radioresistance in numerous NSCLC cases has made the current therapeutic options yield unsatisfactory outcome (Yin et al., 2011). Accumulating studies have reported the crucial role of miRs in the regulation of the malignant development of NSCLC through their roles as oncogenes or tumor suppressors (Iqbal et al., 2019; Weidle et al., 2019). However, the specific mechanism of miR-320a in radioresistance of NSCLC remains unclear. Therefore, we aimed to explore the role of miR-320a/HIF1α/KDM5B/PTEN axis in the radioresistance of NSCLC. Collectively, our findings suggested that overexpression of miR-320a resulted in a decrease in radioresistance of NSCLC cells by promoting methylation of PTEN via HIF1α/KDM5B axis suppression (Figure 7).

Firstly, our data revealed the poor expression of miR-320a in NSCLC cells and tissue samples, which promoted radiosensitivity of NSCLC by reducing NSCLC cell proliferation and enhancing apoptosis. Consistent with our study, Kumar et al. (2020) demonstrated that miR-320a was downregulated in the serum of NSCLC patients. Additionally, the poor expression of miR-320 has also been demonstrated in NSCLC tissues and cells (Wang et al., 2017). Further reports by Lv et al. signified that the low expression of miR-320a in lung cancer patients was linked with the short overall survival of these patients (Xing et al., 2018). Nevertheless, it has been indicated that the expression of miR-320a was linearly increased with the radiation dose and treatment duration, which could promote the radiosensitivity of cancer (Hu et al., 2018). Accordingly, overexpressed miR-320a has been reported to inhibit the proliferation and metastasis, while promoting irradiation-induced apoptosis of lung adenocarcinoma cells (Lv Q. et al., 2017). Wan et al. (2015) observed that miR-320a upregulation led to the suppression of the colon cancer cell proliferation and migration, resulting in hypersensitivity to chemoradiotherapy. Intriguingly, our study illustrated the targeting relationship between miR-320a and HIF1α in NSCLC and identified HIF1α as a target gene of miR-320a. In retinoblastoma, it has been reported that miR-320 suppressed autophagy by targeting HIF1α under hypoxic conditions, which was consistent with our findings (Liang et al., 2017).

Subsequent experimental data of our study demonstrated that HIF1α was overexpressed in NSCLC tissues, promoting cell proliferation while inhibiting apoptosis secondary to irradiation, thus supporting the hypothesis that HIF1α overexpression is associated with promoted radioresistance in NSCLC. Accordingly, previous experimental data confirmed that HIF1α is overexpressed in NSCLC cells, which attributed to the high proliferation capacity of NSCLC cells (Ding et al., 2013). Consistently, HIF1α expression is elevated in NSCLC tissues in NSCLC tissues compared with non-cancerous tissues (He et al., 2015). Importantly, inhibition of HIF1 was found to resensitize cancer cells to radiation (Jiang et al., 2016), which further supports the findings in our study. Nevertheless, the stimulating role of HIF1α in radioresistance has been verified in various types of cancers. For instance, HIF1α downregulation contributed to the suppression of radioresistance of glioblastoma cells (Gouaze-Andersson et al., 2016). The promotive role of overexpressed HIF1α in radioresistance has been elaborated in prostate cancer cells, the mechanism of which was found to be by inducing β-catenin nuclear translocation (Luo et al., 2018). The upregulation of HIF1α caused by down-regulation of miR-21 was reported to promote radioresistance of NSCLC (Jiang et al., 2016). Thus, miR-320a overexpression repressed the radioresistance of NSCLC cells by targeting HIF1α.

Notably, our study revealed that HIF1α activated KDM5B which led to further inhibition of PTEN expression through its demethylase function. Consistently, recent studies have indicated that HIF1α increased the expression of histone demethylase KDM5B in cancer cells (Xia et al., 2009; Salminen et al., 2016). Furthermore, KDM5B is known to reduce the expression of PTEN by demethylating H3K4me3 in the promoter region of PTEN in liver cancer (Tang et al., 2015). Moreover, we reported that HIF1α promoted radioresistance of NSCLC cells by decreasing the PTEN expression via KDM5B upregulation. Intriguingly, up-regulated histone demethylase KDM5B has been found in NSCLC cells and was associated with poor survival of patients (Kuo et al., 2018). Another study suggested that overexpression of histone demethylase KDM5B resulted in promoting the radioresistance of lung squamous cell carcinoma (Bayo et al., 2018). Nevertheless, a significant reduction of PTEN has been reported in NSCLC cells whereas increased expression of PTEN was attributed to the development of NSCLC (Yu et al., 2017). Additionally, increased expression of PTEN was found to suppress the radioresistance of NSCLC through the stimulation of miR-21 (Liu et al., 2013). In addition, miR-18a/PTEN axis has been reported to induce the sensitivity of NSCLC cells to cisplatin by stimulating the tumor protein 53 target gene 1 (Xiao et al., 2018). Taken together, our study provided evidence that miR-320a could potentially contribute to the inhibition of radioresistance of NSCLC cells through the regulation of the HIF1α/KDM5B/PTEN axis.

## Conclusion

In summary, the present findings highlighted that an increase in miR-320a resulted in the inhibition of HIF1α and KDM5B expressions. Nevertheless, KDM5B could enhance the radioresistance of NSCLC via the inhibition of PTEN. Moreover, up-regulated miR-320a inhibited the radioresistance of NSCLC by promoting methylation of PTEN via HIF1α suppression, hence, further enhancing the rational basis for its application in the treatment of NSCLC. While our study demonstrated the significant implications for the radioresistance of NSCLC treatment, further investigations are required to elucidate the underlying mechanism mediating the interaction among miR-320a, HIF1α, KDM5B, and PTEN in NSCLC.

## Data Availability Statement

The original contributions presented in the study are included in the article/Supplementary Material, further inquiries can be directed to the corresponding authors.

## Ethics Statement

The studies involving human participants were reviewed and approved by the Ethics Committee of Tianjin Medical University Cancer Institute & Hospital. The patients/participants provided their written informed consent to participate in this study. The animal study was reviewed and approved by the Committee on the Ethics of Animal Experiments of Tianjin Medical University Cancer Institute & Hospital.

## Author Contributions

PW conceived and designed the research. L-MX, Y-JY, HY, JZ, and YM performed the experiments. X-CC, JW, and L-JZ interpreted the results of experiments. L-MX analyzed the data. Y-JY and HY prepared the figures. L-MX, Y-JY, and HY drafted the manuscript. L-MX edited and revised the manuscript. All authors read and approved the final manuscript.

## Conflict of Interest

The authors declare that the research was conducted in the absence of any commercial or financial relationships that could be construed as a potential conflict of interest.

## Footnotes

1 https://www.ncbi.nlm.nih.gov/

2 http://starbase.sysu.edu.cn/index.php

3 http://gepia.cancer-pku.cn/index.html

## Supplementary Material

Supplementary Figure 1 The profiles of dual-luciferase reporter plasmid vectors.

## Abbreviations

ANOVA: analysis of variance
cDNA: complementary DNA
ChIP: Chromatin immunoprecipitation
FITC: fluorescein isothiocyanate
GAPDH: glyceraldehyde-3-phosphate dehydrogenase
GEO: Gene Expression Omnibus
GEPIA: Gene Expression Profiling Interactive Analysis
HIF1 α: hypoxia-inducible factor-1 α
IgG: Immunoglobulin G
Lv: lentiviral vector
miRs: microRNAs
MUT: mutant
NC: negative control
NSCLC: non-small cell lung cancer
oe: overexpression
RT-qPCR: reverse transcription-quantitative polymerase chain reaction
SDS: sodium dodecyl sulfate
si: small interfering
TCGA: The Cancer Genome Atlas
UTR: untranslated region
WT: wild type.
