# Supplementary figures and images for: Overcoming of Radioresistance in Non-small Cell Lung Cancer by microRNA-320a Through HIF1α-Suppression Mediated Methylation of PTEN
Source: Front Cell Dev Biol. 2020 Nov 10;8:553733. doi: 10.3389/fcell.2020.553733 (PMC7693713; doi:10.3389/fcell.2020.553733)

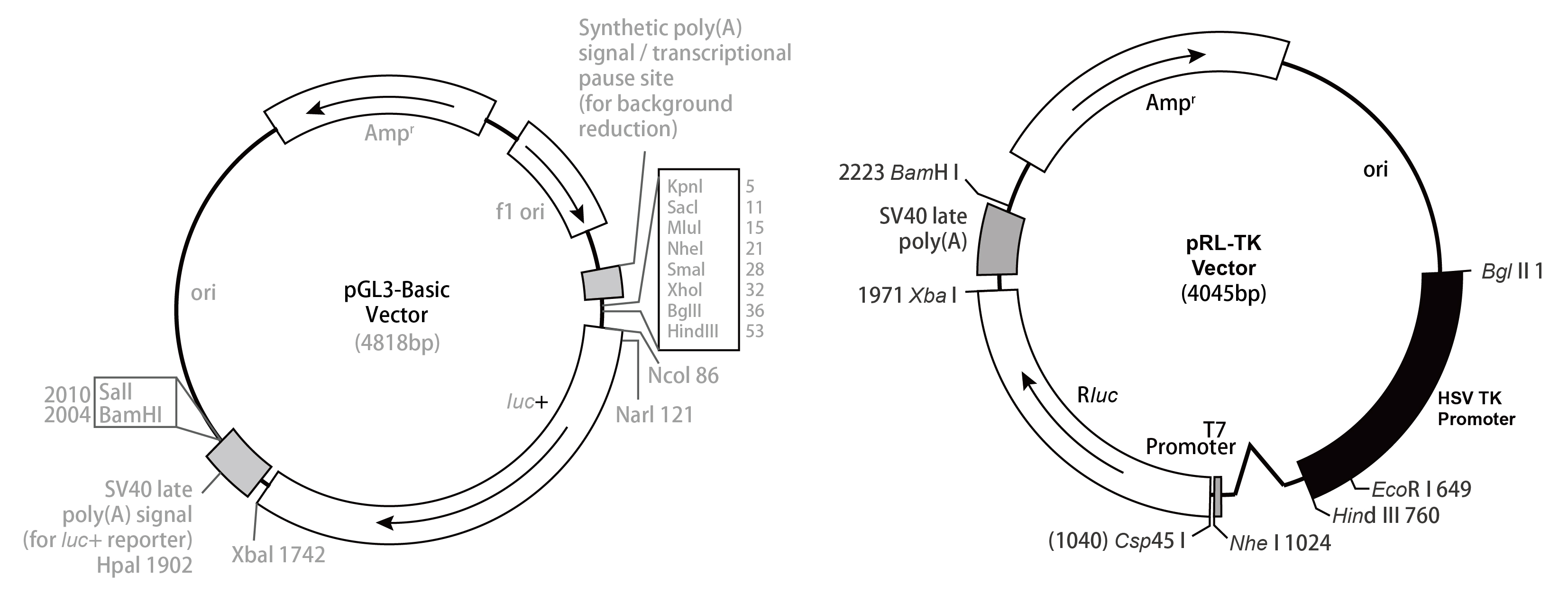

Supplement: Supplementary Figure 1 — The profiles of dual-luciferase reporter plasmid vectors. [file Image_1.JPEG]
